# A Very Rare Case of Primary Renal Squamous Cell Carcinoma Presenting With Xanthogranulomatous Pyelonephritis on Radiological Imaging

**DOI:** 10.7759/cureus.11871

**Published:** 2020-12-03

**Authors:** Bala C Veerabathini, Kaushik Manthani, Sandeep Gandhi, Nerin Duddala

**Affiliations:** 1 Medicine, Peconic Bay Medical Center - Northwell Health, Riverhead, USA; 2 Family Medicine, Peconic Bay Medical Center - Northwell Health, Riverhead, USA; 3 Internal Medicine, Prathima Institute of Medical Sciences, Karimnagar, IND

**Keywords:** pyelonephritis, xanthogranulomatous, kidney, nephrectomy, urinary tract infection, proteus, staghorn calculi, bear paw sign, renal, squamous cell carcinoma (scc)

## Abstract

Xanthogranulomatous pyelonephritis (XGP) and primary renal squamous cell carcinoma (SCC) are both rare conditions that can present very similarly in a patient with renal staghorn calculi. We report a case of a 54-year-old male who presented with fatigue, vomiting and significant acute weight loss. The patient was initially believed to have XGP, but was ultimately diagnosed with both XGP and primary renal SCC. This case report and literature review is meant to elucidate the similarities and differences between XGP and primary renal SCC as both can be diagnostically challenging.

## Introduction

Primary renal squamous cell carcinoma (SCC) of the kidney is an extremely rare and aggressive cancer comprising less than 1% of all malignant renal neoplasms. It presents in men and women equally (particularly 50 to 70 years old) and is usually linked to long-standing nephrolithiasis, especially staghorn calculi. These calculi can result in chronic irritation, inflammation, and/or infection, leading to squamous metaplasia. Other risk factors for squamous metaplasia include vitamin A deficiency, hydronephrosis, smoking, schistosomiasis, chemical use, and hormonal imbalances. Renal SCC can present as large, necrotic, sessile, ulcerated infiltrative masses of the renal parenchyma as well as the perinephric soft tissue [1]. Although the primary SCC of the kidney is rare, it should be considered in patients with longstanding renal calculi, particularly those with large staghorn calculi of the renal pelvis [2]. Tumors of renal SCC show squamous differentiation and can show histological features, including keratin pearl formation, intercellular bridges, and keratotic debris. Renal SCC can be classified into two groups: central and peripheral. Central renal SCC is more intraluminal and is usually found with metastases to the lymph nodes, while peripheral renal SCC is classically in the renal parenchyma and may spread to the perirenal fat before spreading to the lymph nodes or other sites [1, 3].

In contrast, xanthogranulomatous pyelonephritis (XGP), a variant of chronic pyelonephritis, has a prevalence of less than 1 in 10,000 people with a predilection for middle-aged to elderly females. It usually results from obstructing calculi, especially staghorn calculi, and recurrent urinary tract infections. Various bacteria can cause infection/calculi; however, Escherichia coli and Proteus mirabilis are the most frequently isolated organisms [4]. XGP is characterized by diffuse (90% of cases) or focal (10% of cases) severe, renal parenchymal infection resulting in the destruction and replacement of parenchymal cells with lipid-laden macrophages or histiocyte cells, giving the tissue a yellowish color (“xantho” means yellow) and thereby representing a chronic granulomatous disease [4, 5]. In the acute phase of the inflammatory process, neutrophils, lymphocytes, plasma cells, foreign body giant cells, and histiocytes accumulate in the parenchyma. Capillary proliferation and hemorrhage can be present, and the nearby histiocytes engulf the erythrocytes forming the lipid-laden macrophages [6]. The inflammatory process can lead to surrounding inflammation and renal fistulas with nearby structures as well as abscesses [5]. XGP is classified into 3 stages. Stage 1 is where the disease is confined to the renal parenchyma only. Stage 2 involves the renal parenchyma as well as the perirenal fat. Stage 3 is where the disease extends into the perirenal and pararenal spaces or surrounding retroperitoneum [4].

Clinical presentation of both primary renal SCC and XGP can be vague, usually consisting of constitutional symptoms, including malaise, weight loss, and low-grade fever. Flank pain, urinary tract symptoms, hematuria, pyuria, and positive urine cultures can be present but are not always present for both conditions. Renal SCC and XGP can appear similar on imaging as both may present with staghorn calculi, enlarged calyces, and hydronephrosis. Diagnosing renal SCC by imaging can be difficult as no pathognomic features can be seen. However, 18-fluorine fluorodeoxyglucose (18F-FDG) PET/CT imaging is most useful in supporting the diagnosis of renal SCC, with a sensitivity of 62% and a specificity of 88% [1]. For XGP, computed tomography has been the most helpful of the imaging modalities (compared to X-ray and MRI) to support the diagnosis [2, 4, 6]. Contrast-enhanced CT-scan shows the blown-out calyces with cortical thinning, giving the classic “bear paw sign” [6]. For both renal SCC and XGP, histopathology is often used to confirm the diagnosis, and nephrectomy is the definitive treatment for both conditions [2, 5]. While both primary renal SCC and XGP are usually present independently, there have been very few cases documented in the literature with the co-existence of both conditions in patients with chronic nephrolithiasis [7].

## Case presentation

A 54-year-old male landscaper with no significant past medical or surgical history presented to the hospital with fatigue, lightheadedness, decreased appetite, nausea, vomiting, dark stool, and significant weight loss for the one-month duration. He denied headaches, dizziness, night sweats, hemoptysis, shortness of breath, chest pain, constipation, diarrhea, abdominal pain, dysuria, and hematuria in the recent past. The patient denied taking any home medications, including over-the-counter medications, and he denied any history of tobacco, alcohol, or recreational drug use.

He appeared cachectic, fatigued, and was cooperative but in no acute distress. He was conscious, alert, and oriented to person, place, and time. The physical exam was significant for cachexia and abdominal distention but otherwise unremarkable, including a benign cardiovascular, respiratory, abdominal, neurological, and skin exam. His initial vitals were notable for elevated heart rate at 110 beats/minute and body mass index (BMI) of 17.1 kg/m^2^ but were otherwise within normal limits. Pertinent lab results on admission are listed in Table 1. He met systemic inflammatory response syndrome (SIRS) criteria on admission with elevated white blood cell (WBC), elevated heart rate, and elevated lactate. Urinalysis and severe acute respiratory syndrome coronavirus 2 (SARS-CoV-2) Abbott (dry swab) were negative.

**Table 1:** Lab results

	Lab value:	Normal range:
White blood cell (WBC) count	13,490/mm^3^	4,500 to 11,000/mm^3^
Neutrophils, segmented	87.2%	54 to 62%
Neutrophils, banded	11.76%	3 to 5%
Hemoglobin	5.0 g/dL	13.5 to 17.5 g/dL
Hematocrit	16.9%	41% to 53%
Mean corpuscular volume (MCV)	98.8 µm^3^	80 to 100 µm^3^
Mean corpuscular hemoglobin (MCH)	29.2 pg/cell	25.4 to 34.6 pg/cell
Mean corpuscular hemoglobin concentration (MCHC)	29.6 Hb/cell	31 to 36 Hb/cell
Red cell distribution width (RDW)	18.5%	11.5 to 15.4%
Absolute reticulocyte count	164 thousand cells/µL	20 to 80 thousand cells/µL
Platelet count	476,000/mm^3^	150,000 to 400,000/mm^3^
Serum sodium	132 mEq/L	136 to 145 mEq/L
Serum potassium	3.5 mEq/L	3.5 to 5.0 mEq/L
Serum chloride	99 mEq/L	95 to 105 mEq/L
Serum bicarbonate	17 mEq/L	22 to 28 mEq/L
Serum urea nitrogen (BUN)	50 mg/dL	7 to 18 mg/dL
Serum creatinine (Cr)	1.1 mg/dL	0.6 to 1.2 mg/dL
BUN/Cr ratio	45:1	10:1 to 20:1
Protein total	4.4 g/dL	6.0 to 7.8 g/dL
Albumin	2.0 g/dL	3.5 to 5.5 g/dL
Serum lactate level	3.4 mmol/L	.5 to 1 mmol/L
Erythrocyte sedimentation rate (ESR)	83 mm/h	0 to 15 mm/h
C-reactive protein (CRP)	5.09 mg/L	< 3.0 mg/L
Stool occult blood	Positive	Negative

**Figure 1:**
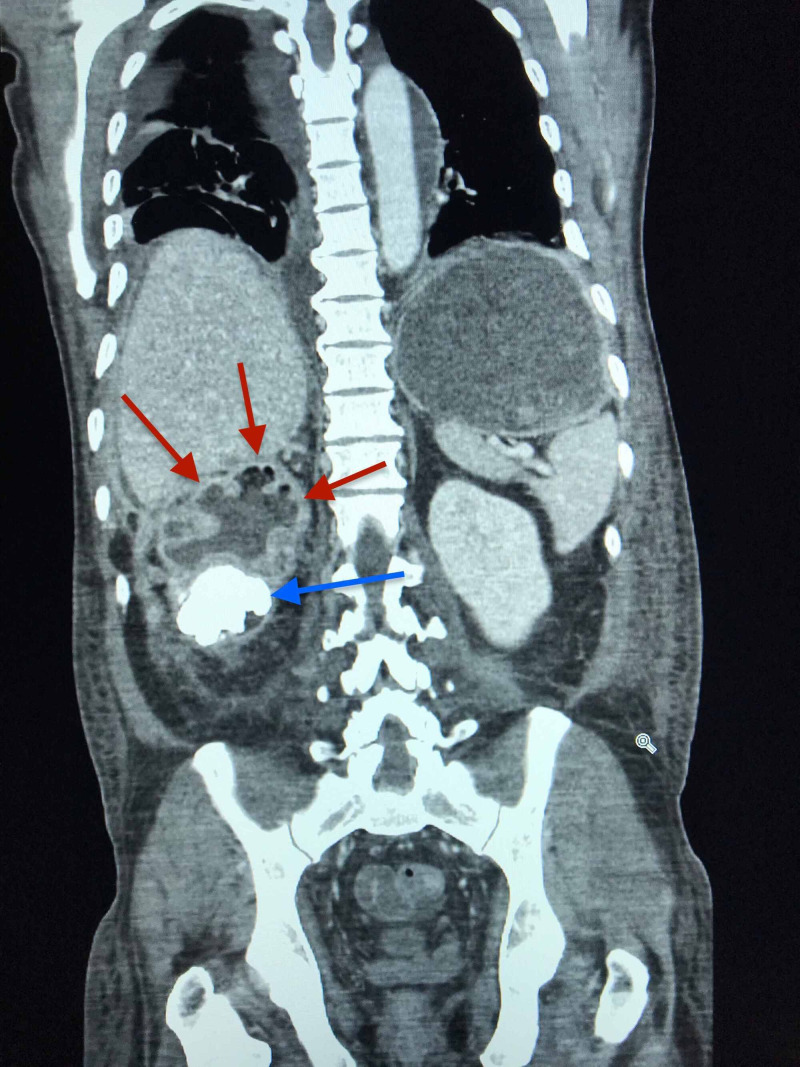
Right kidney visualized with staghorn calculi and dilated renal calyces on CT of abdomen, coronal plane Staghorn calculus is indicated by the blue arrow. Dilated renal calyces are indicated by the red arrows.

**Figure 2:**
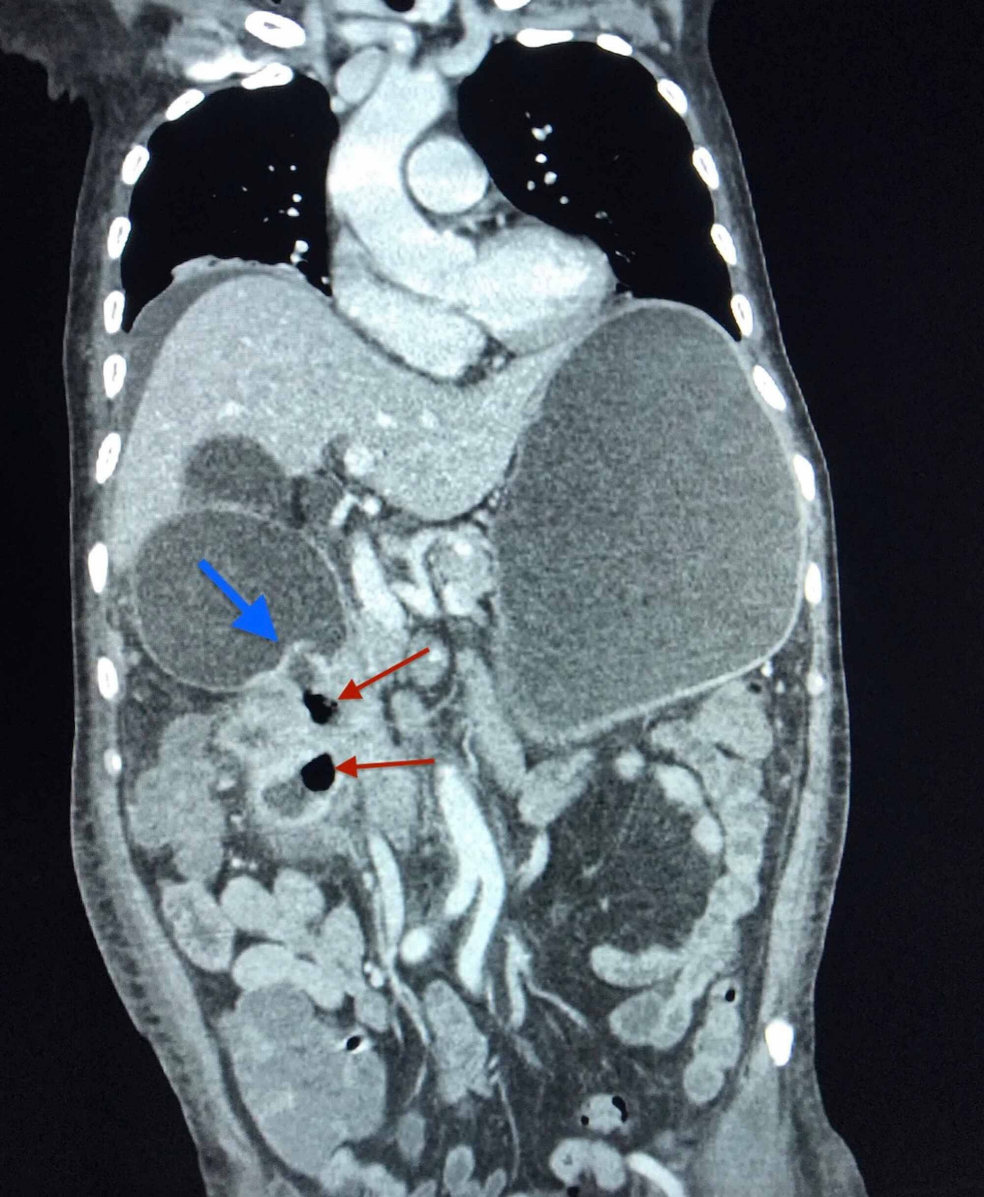
Pyelogastric fistula visualized connecting upper renal calculi with gastric antrum on CT of abdomen, coronal plane Fistula is indicated by the blue arrow. Locules of air are indicated by the red arrows.

**Figure 3:**
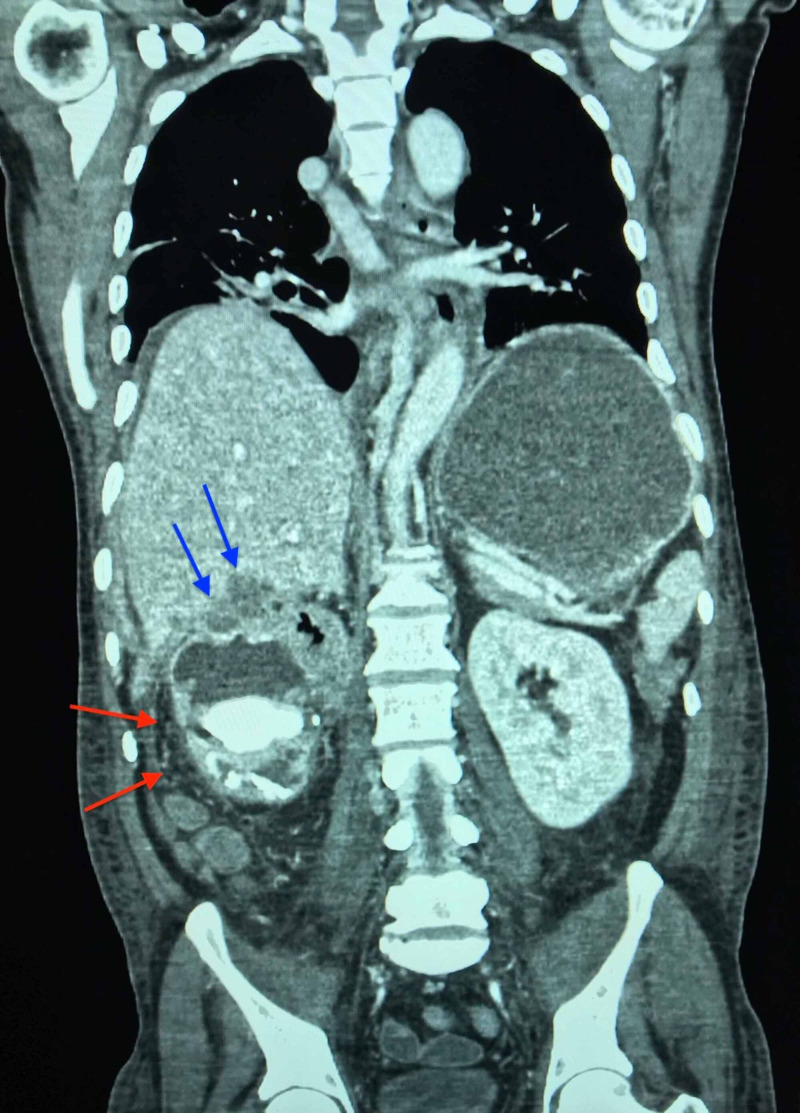
Subscapular hepatic hypodensities and inferior lateral renal densities visualized on CT of abdomen, coronal plane Subscapular hepatic hypodensities are indicated by the blue arrows. Inferior lateral renal nodular densities are indicated by the red arrows along the renal fascia.

**Figure 4:**
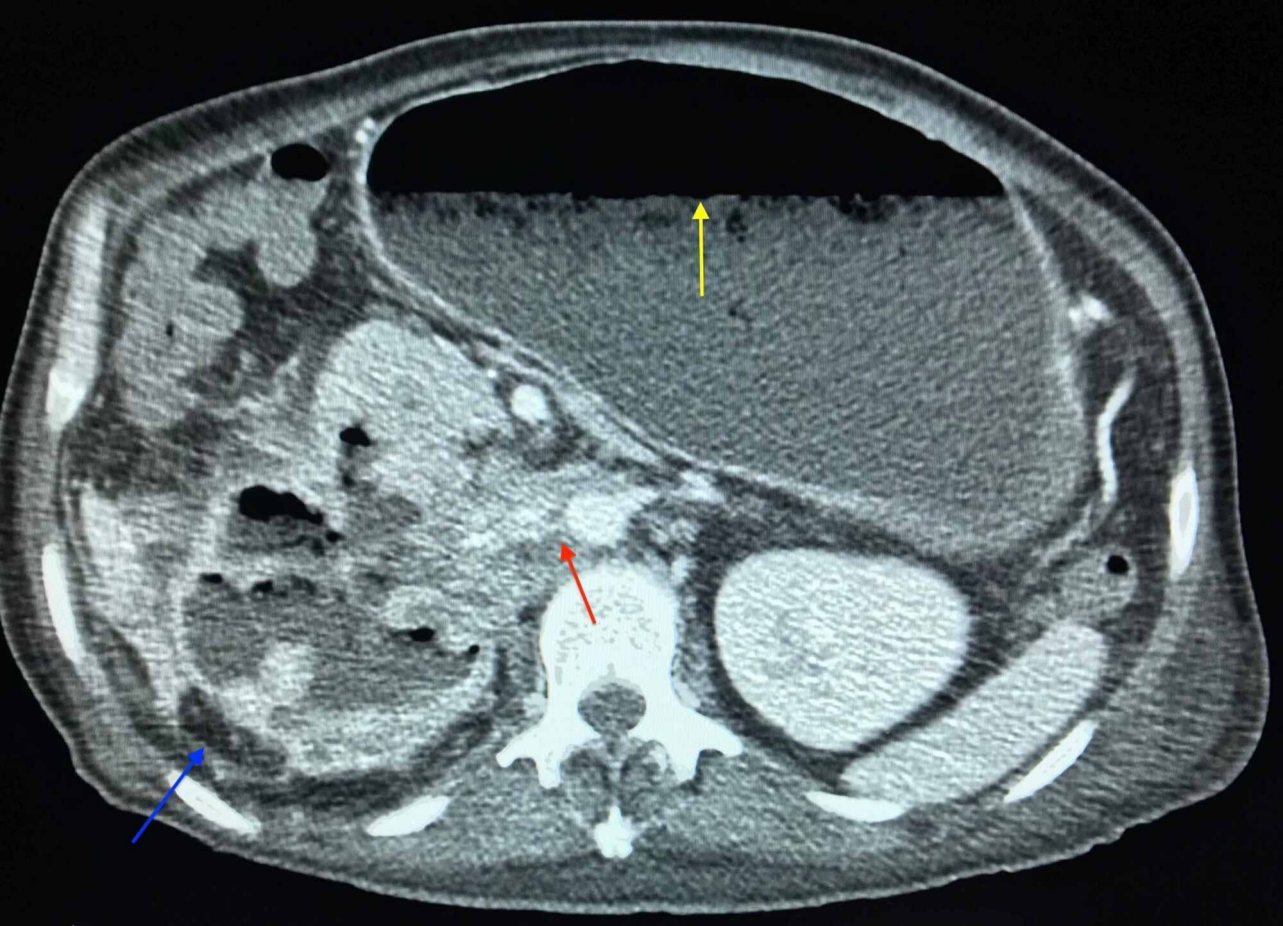
Filling defect of right renal vein/artery and posterior lateral nodular density of right kidney visualized on CT of abdomen, transverse plane Filling defect of right renal vein/artery is indicated by the red arrow. Posterior lateral renal nodular density is indicated by the blue arrow. The air-fluid level of the stomach is indicated by the yellow arrow.

**Figure 5:**
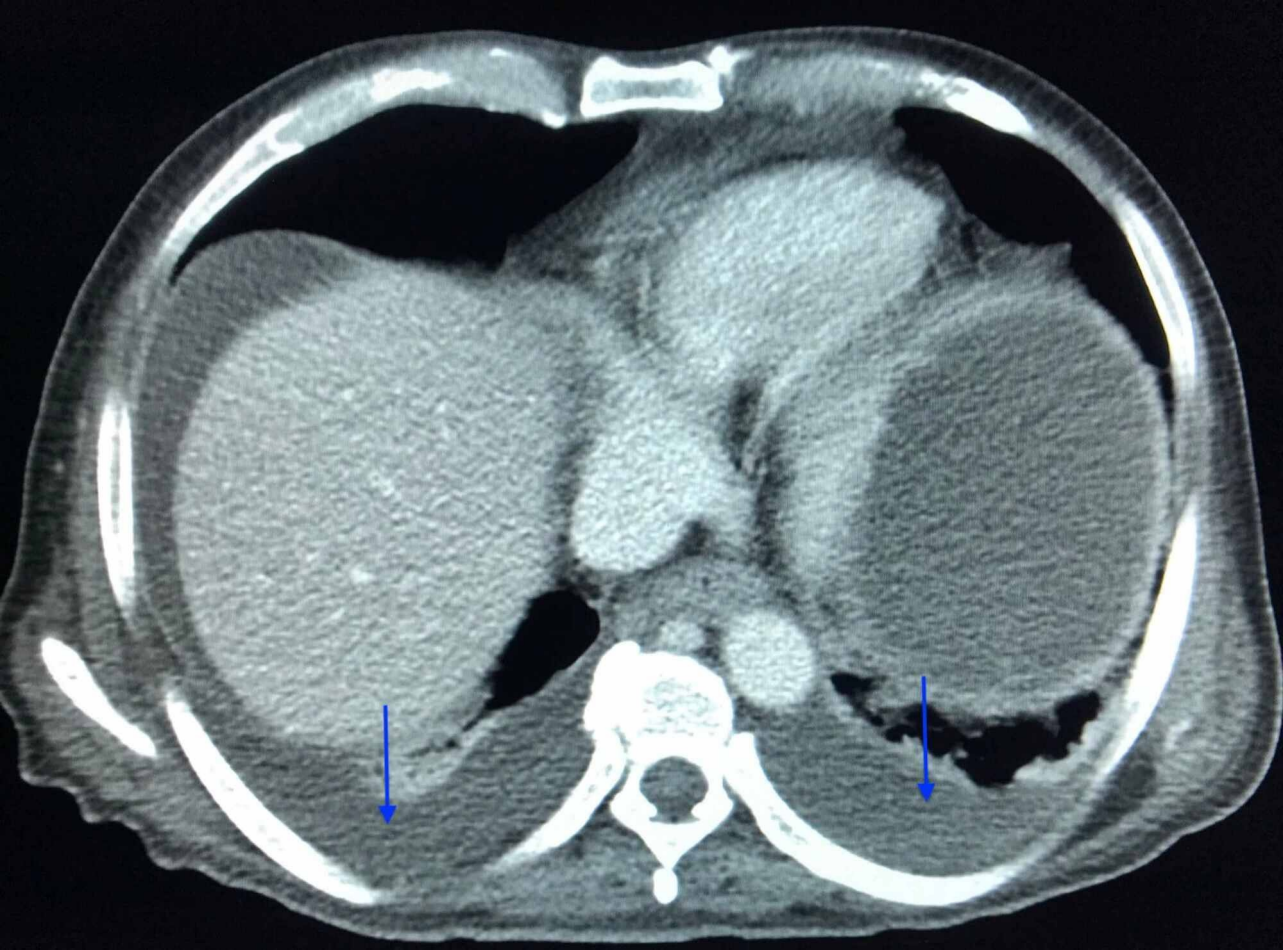
Pleural lung effusions visualized on CT of thorax, transverse plane Pleural effusions are indicated by the blue arrows, as can be seen along the inferior border of the lungs.

CT of abdomen and pelvis with contrast was done, and pertaining results are depicted in Videos 1-3 and Figures 1-5. 

**Video 1:** Abdomen CT with contrast, coronal plane

**Video 2:** Abdomen CT with contrast, transverse plane

**Video 3:** Abdomen CT with contrast, sagittal plane

According to the radiology report, the right kidney was markedly abnormal, demonstrating features suggestive of xanthogranulomatous pyelonephritis, including a large staghorn calculus at the lower pole and diffuse cortical thinning consistent with "bear paw sign" (Figure 1). The right renal cortex was diffusely infiltrated by a heterogenous complex mass, suggestive of possible neoplasm. There was marked hydronephrosis containing locules of air, which appeared to be related to a fistula from the antrum of the stomach to a superior right renal calyx (Figure 2). There was no hydroureteronephrosis and no air seen in the right renal collecting system. Inflammation surrounded the right kidney, and there were some ill-defined, enlarged lymph nodes in the upper right quadrant. Nodular densities suspicious for implants were seen posterior and lateral to the right kidney and along the paracolic gutter, the largest along the facia posterior to the right kidney, and measured up to 1.8 centimeters (Figures 3-4). Additional nodular implants were seen inferior to the right hepatic lobe (Figure 3) with some perihepatic ascites. The nodular implants may be related to the extension of the process in the right kidney. The left kidney appeared within normal limits with no hydronephrosis (Figure 3). The stomach was markedly dilated to the level of the gastric antrum with likely gastric outlet obstruction (Figure 2). The duodenum and small bowel were essentially collapsed. The infrarenal inferior vena cava were not seen, suggesting chronic thrombosis, possibly may be secondary to extension of the mass. The mass was seen encasing and narrowing the superior mesenteric artery and partially encasing the abdominal aorta. The right renal vein and artery were also not readily visualized, presumably thrombosed/obliterated. The portal vein, splenic vein, and superior mesenteric vein were patent, and a prominent azygous vein was noted. A nodular right adrenal gland was seen as well. Moderate pleural effusions could be seen in both lungs (Figure 5).

The patient was given two units of packed red blood cells on admission with intravenous 1 liter sodium chloride 0.9% for over 30 minutes. All electrolytes were repleted as needed. He was put on nil per os (NPO) with intravenous total parenteral nutrition due to gastric outlet obstruction. The patient was transferred to a tertiary care center for further evaluation and treatment. Endoscopy was performed, and the antral and duodenal mucosa biopsy revealed well-differentiated, keratinizing squamous cell carcinoma pathology. The mass involved the antrum and proximal duodenum, adjacent to the fistulous communication area between the right renal collecting system and the gastric antrum. The patient was scheduled for palliative gastrojejunostomy. Unfortunately, the patient decompensated in the following days from complications of the carcinoma and was placed on comfort measures only. The patient expired shortly afterward.

## Discussion

Our patients' radiological findings, including the classical “bear paw sign”, were suggestive of xanthogranulomatous pyelonephritis (XGP). The chronic staghorn calculi made the patient more prone to recurrent urinary tract infections with long-standing pyelonephritis and hydronephrosis. As indicated by the elevated ESR and CRP, chronic inflammatory processes may have led to the pyelogastric fistula and surrounding inflammation. In addition to inflammatory conditions, elevated ESR and CRP may be present in a variety of other conditions, including anemia or cancer.

Our patients' symptoms of fatigue, decreased appetite, and significant weight loss for two months duration were also consistent with carcinoma, which may have spread through the lymphatic and blood system. In addition, the pyelogastric fistula may have led to the dissemination of the carcinoma from the upper pole to the kidney to the gastrum of the stomach and duodenum, suggestive of primary renal squamous cell carcinoma (SCC), especially given the staghorn calculus. Long-term irritation and inflammation induced by staghorn calculi provide a nidus of the cellular changes to metaplasia, dysplasia, and eventually to neoplasia [1, 8]. Patients with renal SCC can present with diverse and non-specific radiologic findings, including an irregular solid mass, hydronephrosis, calcifications, and renal pelvic infiltrative lesions without detecting a distinct mass. The most distinctive findings in CT of renal SCC include an enhancing extra-luminal and exophytic mass, with an intra-luminal component in some cases [8]. The diffuse heterogeneous appearance of the right kidney, as well as the perinephric extension of the nodular densities, may be indicative of the right kidney as the origin of the carcinoma. The masses in the antrum and proximal duodenum may also have led to the small bowel obstruction and resulted in additional symptoms of nausea and vomiting. Since the patients' albumin and total protein were low, the liver may have been affected by the carcinoma as well, as demonstrated by the hepatic nodular densities. This may have led to the pleural effusions in both lungs as well as the perihepatic ascites.

Our patient met SIRS criteria on admission with an elevated white blood cell count, elevated heart rate, and elevated lactemia, most likely due to inflammatory effects resulting from the renal SCC and XPG. Tumors can produce granulocyte colony-stimulating factors (G-CSF), which can skew the neutrophil balance in bone marrow, leading to increased blood neutrophils production by the bone marrow. Neutrophils are an important source of matrix metallopeptidase (MMP-9) in malignancy, which acts to release angiogenic factors, particularly vascular endothelial growth factor (VEGF), from the extracellular matrix [9].

Our patients' elevated absolute serum urea nitrogen (BUN) and BUN/creatinine ratio may have resulted from pre-renal causes or gastrointestinal (GI) bleed, as evident by the positive stool occult and history of dark stools. According to a study conducted in patients with gastrointestinal bleeding, a BUN/creatinine ratio of greater than 35 was indicative of an upper GI bleed. The BUN/Cr ratio correlated significantly with an upper GI source of bleeding (p=0.03), with a ratio greater than 36 having a sensitivity of 90% and a specificity of 27% [10]. Our patients' bleed source was likely to be the gastric antrum or the proximal duodenum at the location of the carcinoma. Since the patient has a normal mean cell volume (MCV), high red cell distribution width (RDW), low mean corpuscular hemoglobin concentration (MCHC), and increased reticulocyte count, the patient may have a chronic iron deficiency anemia superimposed on an acute bleed scenario. The bone marrow may have produced an increased number of immature, large reticulocytes to compensate for the low hemoglobin (resulting in microcytic cells) due to the bleed. As a result, the mean cell volume may be in the normal range, and the red cell distribution width (RDW) may be increased. Since the patients' right kidney was affected by the carcinoma, there is a possibility for decreased erythropoietin (EPO) production from that kidney but with an appropriate compensatory increase in EPO from the healthy kidney for stimulation of bone marrow turnover. 

The overall mortality from XGP is very low. However, morbidity from chronic kidney dysfunction is substantial [11]. Patients with renal SCC usually present in advanced stages, as demonstrated by our case. As a result, the prognosis is poor (5-year survival rate less than 10%) as surgical resection is rarely curative and adjuvant chemotherapy is usually ineffective [12]. Therefore, the decision of palliative care with comfort measures was deemed appropriate before the unfortunate expiration of our patient. 

## Conclusions

The co-occurrence of both primary renal squamous cell carcinoma (SCC) and xanthogranulomatous pyelonephritis (XGP) in the same kidney is rare but possible, as illustrated by this case report. Diagnosis is more readily attainable with the advent of cutting-edge technologies like high-resolution computed tomography and biopsy-related procedures. Despite this, surgery may not provide curative treatment in advanced stages of renal SCC or XGP, and therefore earlier detection of disease processes may prove imperative for such patients. As both XGP and renal SCC are highly morbid conditions, early recognition of such conditions by radiologists is essential for better patient outcomes. 
